# A multi-year data set on aerosol-cloud-precipitation-meteorology interactions for marine stratocumulus clouds

**DOI:** 10.1038/sdata.2018.26

**Published:** 2018-02-27

**Authors:** Armin Sorooshian, Alexander B. MacDonald, Hossein Dadashazar, Kelvin H. Bates, Matthew M. Coggon, Jill S. Craven, Ewan Crosbie, Scott P. Hersey, Natasha Hodas, Jack J. Lin, Arnaldo Negrón Marty, Lindsay C. Maudlin, Andrew R. Metcalf, Shane M. Murphy, Luz T. Padró, Gouri Prabhakar, Tracey A. Rissman, Taylor Shingler, Varuntida Varutbangkul, Zhen Wang, Roy K. Woods, Patrick Y. Chuang, Athanasios Nenes, Haflidi H. Jonsson, Richard C. Flagan, John H. Seinfeld

**Affiliations:** 1 Department of Chemical and Environmental Engineering, University of Arizona, Tucson, Arizona 85721, USA.; 2 Department of Hydrology and Atmospheric Sciences, University of Arizona, Tucson, Arizona 85721, USA.; 3 Division of Geological and Planetary Sciences, California Institute of Technology, Pasadena, California 91125, USA.; 4 Cooperative Institute for Research in Environmental Science and National Oceanic and Atmospheric Administration, Boulder, Colorado 80305, USA.; 5 Division of Chemistry and Chemical Engineering, California Institute of Technology, Pasadena, California 91125, USA.; 6 Chemistry and Dynamics Branch, National Aeronautics and Space Administration Langley Research Center, Hampton, Virginia 23681, USA.; 7 Universities Space Research Association, Columbia, Maryland 21046, USA.; 8 Franklin W. Olin College of Engineering, Needham, Massachusetts 02492, USA.; 9 Environmental Science and Management, Portland State University, Portland, Oregon 97207, USA.; 10 School of Earth and Atmospheric Sciences, Georgia Institute of Technology, Atlanta, Georgia 30332, USA.; 11 School of Chemical and Biomolecular Engineering, Georgia Institute of Technology, Atlanta, Georgia 30332, USA.; 12 Department of Marine, Earth, and Atmospheric Sciences, North Carolina State University, Raleigh, North Carolina 27695, USA.; 13 Environmental Engineering and Earth Sciences, Clemson University, Clemson, South Carolina 29625, USA.; 14 Department of Atmospheric Science, University of Wyoming, Laramie, Wyoming 82071, USA.; 15 Civil and Environmental Engineering Department, University of California-Davis, Davis, California 95616, USA.; 16 Naval Postgraduate School, Monterey, California 93943, USA.; 17 Earth and Planetary Sciences, University of California-Santa Cruz, Santa Cruz, California 95064, USA.; 18 Institute for Environmental Research and Sustainable Development, National Observatory of Athens, Palea Penteli GR-15236, Greece.; 19 Institute of Chemical Engineering Sciences, Foundation for Research and Technology Hellas, Patras GR-26504, Greece

**Keywords:** Atmospheric chemistry, Atmospheric dynamics, Environmental chemistry

## Abstract

Airborne measurements of meteorological, aerosol, and stratocumulus cloud properties have been harmonized from six field campaigns during July-August months between 2005 and 2016 off the California coast. A consistent set of core instruments was deployed on the Center for Interdisciplinary Remotely-Piloted Aircraft Studies Twin Otter for 113 flight days, amounting to 514 flight hours. A unique aspect of the compiled data set is detailed measurements of aerosol microphysical properties (size distribution, composition, bioaerosol detection, hygroscopicity, optical), cloud water composition, and different sampling inlets to distinguish between clear air aerosol, interstitial in-cloud aerosol, and droplet residual particles in cloud. Measurements and data analysis follow documented methods for quality assurance. The data set is suitable for studies associated with aerosol-cloud-precipitation-meteorology-radiation interactions, especially owing to sharp aerosol perturbations from ship traffic and biomass burning. The data set can be used for model initialization and synergistic application with meteorological models and remote sensing data to improve understanding of the very interactions that comprise the largest uncertainty in the effect of anthropogenic emissions on radiative forcing.

## Background & Summary

Interactions among aerosol particles, meteorology, and warm clouds remain poorly understood yet represent an area of intense research owing to their significance for the hydrological cycle, radiative forcing, weather, visibility, and geochemical cycling of nutrients^1^. The representation of microphysical and macrophysical processes relating aerosol particles, clouds, precipitation, dynamics, and thermodynamics in current general circulation models relies on parameterizations that are highly uncertain^2^. Barriers to calculating robustly these interactions include the wide range of length scales (~10^13^ m) they operate on, from a single particle to synoptic scale systems, the complexity of cloud systems and associated feedbacks, the strong coupling between aerosol particles and meteorology, and the inhomogeneous spatial distribution and short lifetime of particles^3^. While particles directly reflect and absorb solar radiation, they indirectly influence the planet’s energy balance via their role in modulating cloud properties^4–6^. This indirect effect is linked to the largest source of uncertainty in estimates of the total anthropogenic radiative forcing^7^. Furthermore, much of this uncertainty focuses on marine stratocumulus clouds that exert a strong negative radiative effect and are the dominant cloud type based on global area^8^.

Observational studies of stratocumulus clouds typically rely on some combination of surface, airborne, and space-borne platforms, with each providing unique benefits and limitations^9^. The primary links between aerosol particles and clouds, and their coupling with thermodynamics and dynamics, occurs at spatiotemporal scales ideal for aircraft^10^. One of the most significant challenges in the aerosol-cloud-climate field of research is untangling the effect of meteorology and aerosol particles on clouds^11^. This requires extensive statistics to analyze how a perturbation in a single parameter of interest leads to a cloud response, which involves holding other parameter values fixed. For example, it is common to analyze field data at a fixed value for a cloud macrophysical parameter such as cloud thickness or liquid water path (LWP; see Table 1 for acronym and variable definitions) with the aim of extracting a statistically significant relationship between aerosol number concentration and a cloud property of interest, such as cloud drop effective radius, cloud albedo, or precipitation rate^12–14^. However, even in these cases, it is cautioned that statistical correlations cannot prove causality, and that synergistic inclusion of advanced cloud models is required^15^.

While significant attention has been given to studying the effects of aerosol particles on clouds and precipitation, an area of research that remains understudied with field data is the effects of clouds and precipitation on aerosol particles. Aerosol particles that enter a cloud either activate into cloud droplets or exist as interstitial aerosol particles. As a result of collisions between interstitial particles and droplets, coalescence among droplets and aqueous-phase chemistry in droplets modify the composition and size distribution of particles in clouds, in turn, altering how particles interact with light and water vapor^16^. Processing through collisions and coalescence alters only the number and size of aerosol particles, while chemical processing affects their composition and size distribution as a result of aqueous-phase reactions that produce low-volatility species that can remain in the aerosol phase after subsequent droplet evaporation. Removal of particles via precipitation is important to understand, as it affects the spatial and vertical distribution of aerosol particles, especially cloud condensation nuclei (CCN), in the atmosphere^17^. Failure to account for wet scavenging effects on aerosol particles below clouds can bias investigations of aerosol effects on clouds that rely on a measurement of sub-cloud aerosol^18^.

The goal of this work is to present a unique data set incorporating measurements from six summertime aircraft campaigns focused on the northeastern Pacific Ocean where a persistent summertime stratocumulus deck exists. The study region is an ideal natural laboratory for investigating aerosol-cloud-precipitation-meteorology interactions due to strong aerosol perturbations from ship emissions^19^ and, sometimes, biomass burning. This data set is especially useful in efforts to improve process-based understanding of cloud and precipitation formation by considering appropriate feedbacks across sub-grid scales, that need to be understood to improve the spatial resolution of large-scale models^11^.

## Methods

### Platform and Campaigns

**Figure 1:**
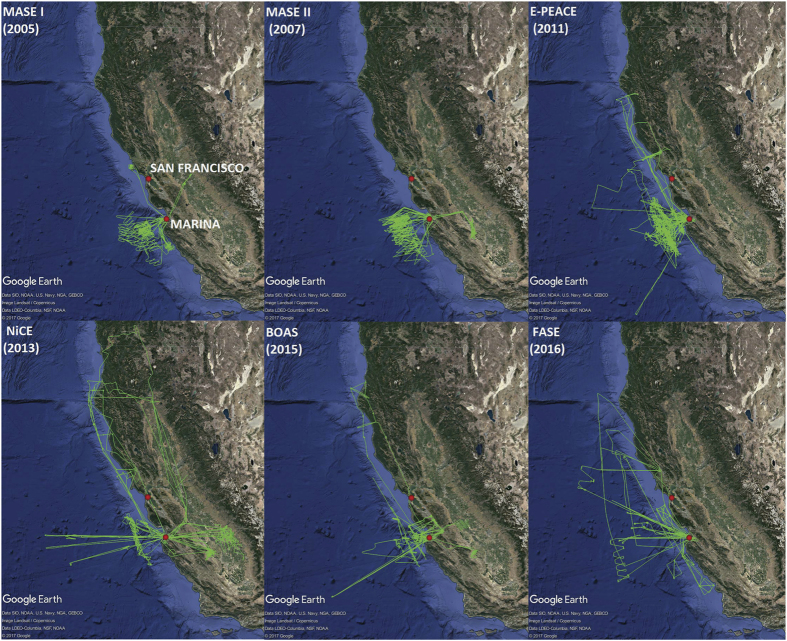
Spatial map summarizing flight tracks for each of the six field campaigns.

The data set presented is based on measurements conducted with the Center for Interdisciplinary Remotely-Piloted Aircraft Studies (CIRPAS) Twin Otter based out of Marina, California. Dates and flight times are summarized in Table 2 (available online only) for each of the following six campaigns: the two Marine Stratus/Stratocumulus Experiments (MASE I, MASE II), the Eastern Pacific Emitted Aerosol Cloud Experiment (E-PEACE), the Nucleation in California Experiment (NiCE), the Biological and Oceanic Atmospheric Study (BOAS), and the Fog and Stratocumulus Evolution Experiment (FASE). The aircraft typically flew at ~55 m s^−1^, with flights ranging from one hour to five hours. The average take-off and landing times were approximately 17:30 UTC (local time=UTC – 7 h) and 21:30 UTC, respectively, with the earliest take-off and latest landing being 14:38 UTC and 02:27 UTC, respectively. Flight tracks for the 113 flight days, which combine for 514 h of total flight time, are shown in Fig. 1. Of special note is that during E-PEACE, an instrumented research vessel (R/V Point Sur) coordinated measurements of aerosol and environmental parameters below the Twin Otter. The R/V Point Sur also executed controlled emissions of smoke from its deck to provide a unique identifiable tracer signature. The cruise lasted from 12-23 July 2011; data from ship-borne measurements can be found elsewhere^20^.

Occasionally, up to three sub-flights were conducted that involved re-fueling at other sites or at Marina. Such days are counted as a single flight in Table 2 (available online only) but are labeled with extensions ‘A’, ‘B’, and ‘C’ for successive flights on a particular day. The objective of multi-flight days was either to capture diurnally-relevant atmospheric features, or to sample in an area that extended outside the range that one flight would allow.

### Flight Strategies

**Figure 2:**
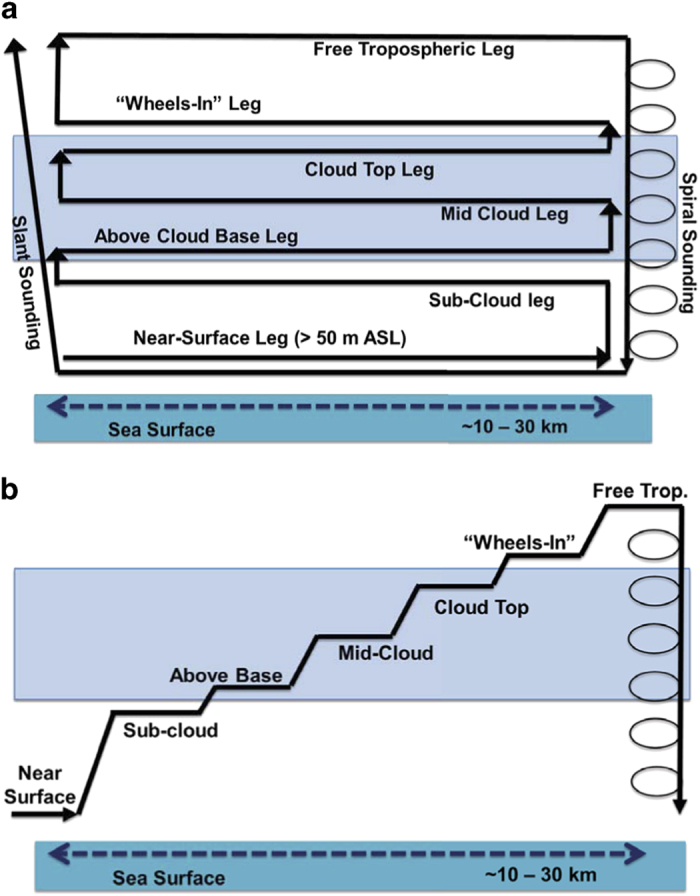
Illustration of two common flight strategies used to probe aerosol-cloud-precipitation-meteorology interactions with the Twin Otter.

**Figure 3:**
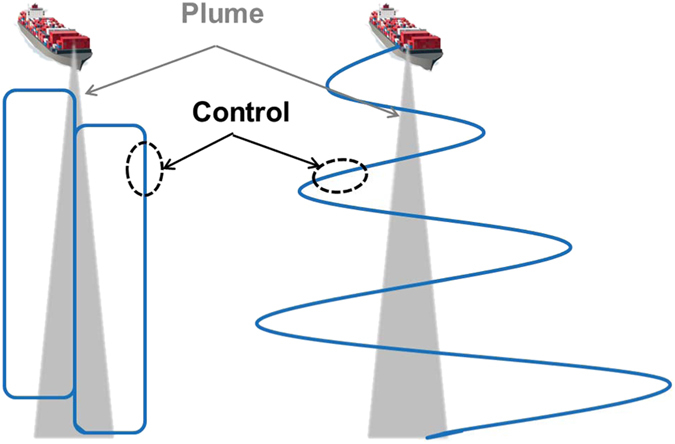
Illustration of two flight strategies used to characterize ship plumes. Blue lines represent the flight track.

The general flight strategy for sampling aerosol and clouds comprised the maneuvers shown in Figs 2 and 3, which excludes the transits between the measurement site and the Marina airport. When the aircraft reached the area of interest after transits, it usually collected data along level legs at multiple altitudes extending from near the ocean surface up to a few hundred meters above cloud top (Fig. 2a). Soundings were conducted periodically throughout the flight in either a slant or spiral maneuver. As some flights involved sampling at distances farther away from the coastline to the west or farther north/south as compared to Marina, another flight strategy comprised stair-step patterns conducted repeatedly until the aircraft turned back on a reverse course to repeat the maneuvers again until reaching the Marina airport (Fig. 2b). Owing to the importance of ship emissions in the study region, the Twin Otter also executed flights paths to characterize the aerosol properties close to the ocean surface as depicted in Fig. 3, prior to repeating the same patterns at higher levels in and above clouds. Data from such maneuvers are useful to contrast the aerosol and cloud characteristics in and out of regions influenced by plumes.

### Instrument Descriptions

Table 3 summarizes the instruments used in each field campaign along with corresponding size and time resolution details. Below we describe the instruments in more detail. Additional guiding details about using data from these instruments, including accuracy, precision, and working ranges can be found in the ‘ReadMe.doc’ file accompanying the data set (Data Citation 1).

### Navigational/Meteorological

Data are presented as a function of UTC time. Standard navigational and meteorological data are provided at 1 Hz time resolution. For specialized analyses though, GPS and thermodynamic data can be provided upon request with 10 and 100 Hz resolution, respectively. The Systron Donner C-MIGITS-III GPS/INS system provided latitude, longitude, and altitude data. Pressure altitude was also determined by use of measurements from a barometric pressure sensor (Setra Model 270). The pressure altitude measured with the Setra sensor is based on static pressure measurements and assumes standard atmosphere. The Setra sensor was plumbed to a static port on the aircraft that has been extensively characterized for location error correction and for dependency on pitch angle and aircraft speed by use of a trailing cone method^21^.

Four differential pressure transducers (Setra Model 239) and two barometric pressure transducers (Setra Model 270), plumbed to a five-hole radome gust probe provided measurements for determination of turbulence and three dimensinoal winds. Horizontal and vertical winds were calculated from these measurements in combination with platform velocity and altitude measurements provided by the C-MIGITS-III GPS/INS system.

A Rosemount Model 102 total temperature sensor provided total temperature measurements, from which ambient air temperature was calculated after taking into account dynamic heating and an instrument-based recovery factor. Humidity data were obtained with an EdgeTech Vigilant chilled mirror hygrometer (EdgeTech Instruments, Inc.). The measurement of dew point temperature by the Edgetech chilled mirror dew point sensor was calibrated using a dew point generator (LI-COR, Inc.). Although not provided, relative humidity (RH) can be calculated based on the ratio of the partial pressure of water vapor relative to equilibrium vapor pressure, both of which are derived from measurements of temperature and dew point temperature. Dew point temperature can be used to calculate specific humidity and water vapor mixing ratio. Furthermore, potential temperature (*θ*) can be calculated from total temperature, while equivalent and virtual potential temperature (*θ*_*e*_ and *θ*_*v*_), additionally require dew point temperature in their calculation. Virtual temperature (*T*_*v*_) can be calculated from total temperature with a correction based on dew point temperature to eliminate the influence of water vapor. Dry air density can be calculated from total temperature and static pressure.

Earth’s skin surface temperature (SST) was measured using a nadir-facing infrared radiation pyrometer (Heitronics KT 19.85). These measurements provide sea surface temperature when the column below the aircraft is clear, or cloud top temperature when the aircraft is above clouds. The pyrometer operates in an infrared spectral range where absorption by CO_2_ and water vapor is minimal, which minimizes errors in the surface temperature measurement.

True air speed (TAS) of the aircraft was determined using measurements of dynamic pressure and temperature, the latter of which was corrected for the Mach number of the aircraft. Dynamic pressure was obtained as the difference between total pressure (from center hole on radome) and static pressure (from static port). An independent measurement of total pressure was also obtained from a pitot tube for validation of the center hole pressure measurement.

### Inlets

Aerosol measurements during the field campaigns were conducted with a forward-facing sub-isokinetic inlet, which samples aerosol below 3.5 μm diameter with 100% efficiency^22^. However, when in cloud, some aerosol instruments were switched via a valve to sample downstream of a counterflow virtual impactor (CVI) inlet. The CVI preferentially samples cloud droplets (rejecting smaller aerosol particles), and then dries them to leave droplet-residue particles for downstream instruments to characterize. During MASE I, the CVI employed was an early version^23^ that was replaced starting in E-PEACE with a new version offering higher sample flow rates from which an increased number of downstream instruments could sample^24^. The previous and current version of the CVI had cutpoint sizes of approximately 10 and 11 μm, respectively. Due to uncertainty in the transmission efficiency of the CVI inlet, data collected downstream of this inlet should be used only to assess relative rather than absolute concentrations, i.e., to determine ratios of relevant parameters. In addition, during MASE II, a rear-facing inlet was also employed in cloud to preferentially sample only interstitial aerosol (i.e., particles that did not activate into cloud droplets). A summary of which instruments sampled downstream each of these three inlets is provided in Table 4.

### Cloud Measurements

Several cloud probes characterized the drop distribution from 0.5 to 1600 μm diameter. The Cloud, Aerosol, and Precipitation Spectrometer (CAPS; Droplet Measurement Technologies, Inc.) is comprised of the Cloud and Aerosol Spectrometer^25^ (CAS; *D*_*p*_~1–61 μm) and the Cloud Imaging Probe (CIP; *D*_*p*_~25–1600 μm). Only data from the forward scattering section of the CAS data (called CASF) are reported. CAPS probe data are converted into size distributions, although it should be noted that the CAS size exhibits considerable uncertainty in the 1-10 μm diameter range owing to influence from Mie resonances. The range of diameters in this size range can vary by a factor of two since drops having different diameters produce similar scattered pulse heights. Data above 10 μm have reduced Mie oscillation contamination, and their uncertainty drops significantly to ~30%^26^.

Other probes providing supporting measurements of drop distribution data included the Cloud Droplet Probe^27^ (CDP; *D*_*p*_~2–52 μm; Droplet Measurement Technologies (DMT), Inc.) and Forward Scattering Spectrometer Probe (FSSP; *D*_*p*_~2–45 μm; Particle Measuring Systems (PMS), Inc., modified by DMT, Inc.). The cloud probes were calibrated using standard methods including with monodisperse polystyrene and glass beads. Past work has discussed uncertainties in counting and sizing associated with these instruments^25,27,28^. CASF, CDP, and FSSP data are useful for quantification of cloud droplet number concentration (*N*_*d*_), droplet effective radius (*r*_*e*_), liquid water content (LWC), and cloud optical depth^29^, while the CIP is most useful for quantification of precipitation rates using documented methods, such as those relating drop size and fall velocity^30,31^. Users should refer to literature to determine size thresholds for cloud droplets versus drizzle droplets^32,33^. The number concentrations reported for each size bin can be added to determine total drop concentrations from each probe across the size range of interest. It is cautioned that the smallest bin channel for all probes is subject to uncertainty due to poorly defined lower and upper limit diameters for those bins.

The PVM-100 A probe^34^ provides a separate measurement of LWC that does not require integrating cloud drop size distributions. Cloud LWC is important for a number of other reasons, such as providing a way to quantify cloud adiabaticity and cloud liquid water path (LWP)^29^, and identifying the cloud-base and cloud-top, based on threshold values chosen by the data user^35–37^. However, users may instead prefer to use the cloud probe data described previously to quantify values of the aforementioned cloud properties. The sensitivity of the PVM-100 A probe decreases in drizzle, as it is designed to respond to droplets smaller than 50 μm^38^. Thus, the use of LWC data requires caution for precipitating conditions.

Dissolved non-water constituents of cloud water were speciated and quantified using samples collected with a modified Mohnen cloud water collector^39^. This collector was deployed in four of the six campaigns, starting with E-PEACE. When in cloud, this slotted rod collector was manually extended out of the top of the aircraft; samples were collected in high-density polyethylene bottles, typically for 5–30 min. Each liquid sample was then split into a number of fractions for different types of analyses: (i) pH (Oakton Model 110 pH meter that was calibrated with 4.01 and 7.00 pH buffer solutions for E-PEACE, NiCE, and BOAS; Thermo Scientific Orion 8103BNUWP Ross Ultra Semi-Micro pH probe for FASE); (ii) water-soluble ionic composition (Ion Chromatography, IC; Thermo Scientific Dionex ICS – 2100 system); and (iii) water-soluble elemental composition (Inductively Coupled Plasma Mass Spectrometry, ICP-MS: Agilent 7700 Series for E-PEACE, NiCE, and BOAS; Triple Quadrupole Inductively Coupled Plasma Mass Spectrometry (ICP-QQQ; Agilent 8800 Series) for FASE). Liquid concentrations were converted into air-equivalent concentrations via multiplication with the average LWC during sample collection.

### Aerosol Measurements

Particle concentrations were recorded in each campaign using multiple condensation particle counters (CPCs; TSI Inc.), specifically a CPC 3010 (D_p_>10 nm) and ultrafine CPC (UFCPC) 3025 (D_p_>3 nm). The saturation thresholds of these two CPCs are 10^4^ and 10^5^ cm^−3^, respectively; concentrations above those limits are subject to coincidence errors, so use of such data is discouraged. Aerosol size distributions were obtained in each campaign with a Passive Cavity Aerosol Spectrometer Probe (PCASP; PMS, Inc., modified by DMT, Inc.; 0.1–2.6 μm) and a Scanning Mobility Particle Sizer (SMPS; ~10–800 nm), which is comprised of a differential mobility analyzer (DMA; TSI Inc. Model 3081) coupled to a model 3010 TSI CPC. Particle sizing by the SMPS was calibrated using polystyrene latex spheres (PSLs), while the PCASP was calibrated with PSLs, water, and dioctyl sebacate. A reference CPC (TSI 3010) was used to calibrate the concentration performance of both the SMPS and PCASP.

Submicrometer aerosol composition was measured with multiple instruments. The first instrument was the Aerosol Mass Spectrometer (AMS; Aerodyne Research Inc.), which quantified non-refractory aerosol composition including sulfate, nitrate, ammonium, chloride, and organics^40–43^. A Compact Time-of-Flight AMS (C-ToF-AMS) was used in MASE I, MASE II, E-PEACE, and NiCE, whereas a High Resolution Time-of-Flight AMS (HiRes-ToF-AMS) was used in BOAS. At the entrance of the AMS, an aerodynamic lens focuses aerosol with vacuum aerodynamic diameters between approximately 50 and 800 nm through a chopper and onto a vaporizer (~600° C). Upon vaporization, molecules undergo electron impact ionization and the resulting ions are detected by a time of flight mass analyzer. A pressure-controlled inlet maintained the sample flow rate at~1.4 cm^3^ s^−1^ to the AMS vacuum chamber. The AMS ionization efficiency (ratio of molecules ionizing relative to total molecules entering instrument) was calibrated prior to flights using dried ammonium nitrate particles. Data are reported for bulk aerosol based on ensemble average mass spectra. Spectra were analyzed in IGOR Pro (WaveMetrics, Inc.) based on SQUIRREL and PIKA modules. Data were corrected for gas-phase interferences using a fragmentation table^44,45^. Composition-dependent collection efficiencies were quantified and applied using a widely used approach^46^ beginning with E-PEACE, while before that (MASE I/II) the collection efficiency was estimated for each flight based on matching the total AMS mass to the mass determined from the SMPS multiplied by the density derived from a comparison of size distributions form the AMS and the SMPS. The two methods yield similar estimates of the collection efficiency for particles measured in this region^42^.

Water-soluble ionic composition was measured with a Particle-Into-Liquid Sampler (PILS; Brechtel Manfucaturing Inc.) coupled to off-line ion chromatography (IC) analysis^47^. More specifically, vials on a rotating carousel collected samples every ~5 min, the contents of which were processed with IC after each flight. The PILS-IC technique speciated a suite of inorganic species (chloride, nitrite, bromide, nitrate, sulfate, sodium, ammonium, magnesium, calcium), amines (ethylamine, dimethylamine, diethylamine), and organic acids (acetate, glycolate, formate, pyruvate, glyoxylate, maleate, oxalate, malonate, succinate, glutarate, adipate, suberate, azelate, methanesulfonate). Three denuders were used to minimize biases associated with acidic and basic inorganic gases, or with volatile organic compounds (VOCs). The instrument’s droplet impactor plate was routinely cleaned between aircraft flights.

A focus during the 2015 BOAS campaign was on biological particles. A Model 4 Waveband Integrated Bioaerosol Sensor (WIBS-4, DMT, Inc.) was deployed to detect and quantify primary biological aerosol particle (PBAP) loading. WIBS-4 measures particle light scattering and autofluorescence of individual particles with diameters between 0.5 and 16 μm. Particles are initially sized using the 90° side-scattering signal from a 635 nm continuous-wave diode laser. The scattering intensity is directly related to particle diameter, subject to Mie resonances as with the other optical probes; it was calibrated prior to deployment using PSL calibration standards (0.8, 0.9, 1.0, 1.3, 2.0, 3.0 μm diameter, Thermo Scientific Inc.). WIBS-4 optical sizing is, therefore, based on the PSL refractive index of 1.59. Successive pulses of 280 nm and 370 nm xenon flashtube ultraviolet light excites chromophores within the PBAP (e.g., Tryptophan, Tyrosine, Riboflavin), with the fluorescence from each successive excitation pulse captured by two fluorescence detectors (FL1, FL2). From the resulting autofluorescence, three important fluorescent emissions are measured: (i) FL1_280 refers to the detected emission between 310-400 nm after excitation at 280 nm; (ii) FL2_280 refers to the detected emission between 420-650 nm after excitation at 280 nm; and (iii) FL2_370 refers to the detected emission between 420-650 nm after excitation at 370 nm. The resulting autofluorescence from 280 nm excitation is mainly linked to the presence of Tryptophan, and the resulting autofluorescence from the 370 nm excitation is linked to the presence of riboflavin and co-enzyme Nicotinamide Adenine Dinucleotide Phosphate (NAD(P)H) within the cells^48^. The WIBS-4 was connected to a laminar flow box to minimize flow rate variability across different altitudes. Subsequently, the volumetric flow rate of the WIBS-4 was adjusted to 2.3 LPM, splitting the main flow to a 1.3 LPM sheath flow and a 1.0 LPM sample flow. Preceding each BOAS flight, a Force Trigger calibration was performed for five minutes to determine the background autofluorescence for each channel (FL1_280, FL2_280, FL2_370). FT calibrations measured the background autofluorescence of particle-free air (no air flow in WIBS-4) and recorded the fluorescence intensity in each channel for 500 excitation flash events^49^.

During E-PEACE, 1 Hz refractory black carbon (BC) measurements were conducted with a Single Particle Soot Photometer (SP2; DMT, Inc.). The data reported include BC number concentration and mass concentration, with all operational and calibration details summarized elsewhere^50^. The main distinction from the previous SP2 deployment discussed in that study is the working range; during E-PEACE, single BC-containing particles were measured with a refractory mass between about 0.5 and 100 fg, or approximately 83-478 nm volume-equivalent diameter. BC number and mass concentrations are reported for the working range of the SP2 instrument only and are not corrected for any BC mass occurring outside the limits of detection, which could increase mass concentration by 15-20% in some environments^50^. The uncertainty in reported mass is ~40% because of limitations of BC standards that are available for calibration.

Measurements of aerosol hygroscopicity were conducted for the super-saturated regime. Cloud condensation nuclei concentrations at supersaturations between 0.1 and 0.85% were obtained with a streamwise thermal-gradient CCN counter^51,52^ (CCNC; DMT, Inc.). The instrument pressure was maintained at 700 mb independent of ambient pressure using a flow orifice and active control system^42^. In most campaigns, a single column CCNC was used, but starting with NiCE, a dual column version was used to quantify CCN concentrations at two supersaturations simultaneously. The instrument was calibrated using documented methods^53^. If of interest to users, scanning flow mode operation^54^ was also used to provide CCN spectra over 20–40 s intervals, with those data available upon request.

In-cloud aerosol data must be used with caution for any instrument that was not sampling downstream of the CVI or rear-facing inlet (Table 4). This applies especially to the PCASP, as it was on the aircraft wing. These data can be vulnerable to drop shatter effects.

### Gas-Phase Measurements

Gas-phase measurements were conducted during BOAS and FASE. During BOAS only, NO_x_ (NO+NO_2_), O_3_, CO, and CO_2_ were measured. NO_x_ was measured with a Los Gatos Research (LGR, Inc.) NO_2_ Analyzer. A NO_x_-scrubbed ozone-rich controlled mixing flow into the instrument’s inlet stream was used to convert NO to NO_2_. Ozone was measured with a 2B Technologies Ozone Monitor (Model 205 Dual Beam Ozone Monitor). CO and CO_2_ were measured with a LGR CO/CO_2_ Analyzer, which was also used for CO data during FASE. During FASE only, CO_2_ and water vapor measurements were conducted with a LI-7200RS CO_2_/H_2_O Analyzer (LI-COR, Inc.).

## Data Records

Field campaign data files (Data Citation 1) are available in separate folders for each campaign: ‘MASE1’, ‘MASE2’, ‘E-PEACE’, ‘NiCE’, ‘BOAS’, ‘FASE’. A ‘ReadMe.doc’ file is provided separately that applies to files in each of the campaign folders. It specifically provides a description of column headers in each file, appropriate units, and usage guidelines. All files are labeled with the extension ‘V1’, indicative of Version 1, with any subsequent updates in the future labeled as V2, V3, and so forth; specific files that are updated will have a note in the ‘ReadMe.doc’ file specifying what was changed. Specific data files in each folder are in csv file format.

## Technical Validation

The quality of data from each instrument has been assured based on multiple steps. First, each instrument group that participated in the six campaigns conducted quality control based on their respective user community methods. Second, with a few exceptions (WIBS, gas-phase sensors), data from each instrument along with details about the measurements have been documented in peer-reviewed manuscripts for at least one of the campaigns in Table 2 (available online only): Meteorological/thermodynamic sensors^29,35,55^, CAPS/FSSP/CDP cloud probes and PVM-100 A probe^24,29,56–59^, CPCs and PCASP^35,41–43,60^, C-ToF-AMS^41–43,61,62^, HiRes-ToF-AMS^36^, cloud water collector^36,63–66^, PILS-IC^61,67,68^, CCNC^41,61,69^, SP2^50^, SMPS^62,69^, CVI^23,24^, forward-facing inlet^22^.

In the third step, a subset of authors conducted a final quality control, consisting of the following corrections:

1. Replace default error values, unrealistic values, and blanks in the instrument data (e.g., ‘-9999’ is recorded in some instruments when there is an instrument failure) with ‘NaN’ (i.e., not a number).
2. Homogenize the units of the data, e.g., all temperatures are in degrees Kelvin, longitude is in °E, latitude in °N, etc.
3. Correct discontinuities in spatial and temporal coordinates using linear interpolation, namely, UTC, latitude, longitude, C-MIGITS-III altitude, and pressure altitude.
4. Only keep measurements between one minute before takeoff and one minute after landing; takeoff and landing times are listed in Table 2 (available online only). In addition, ensure that all instruments with 1 Hz resolution start and end at the same time and have the same number of rows.
5. Clean the size distribution of the probes by removing rows that are obvious outliers, e.g., when the number count is about the same in all the bins.

The ‘ReadMe.doc’ file (Data Citation 1) summarizes the accuracy, precision, working range, and any other necessary details to allow for proper data usage. In order to make data visualization easier for data users, a Matlab plotting code is included with the data set (Data Citation 1). Refer to the ‘ReadMe.doc’ file for instructions on how to use the visualization tool for any flight(s) of interest.

## Usage Notes

The data sets provided can be used to conduct a wide range of studies, including examining aerosol microphysics, cloud physics and dynamics, aerosol-cloud-precipitation-meteorology interactions, cloud water composition, CCN and cloud drop number concentration closure studies, and validation/improvement studies of satellite retrievals and models of varying spatial scales and complexity.

Users can time synchronize data at 1 Hz resolution to slower instruments such as the AMS or the SMPS for studies requiring various instrument measurements to be directly compared. Detailed studies of clouds may require users to calculate parameters that are beyond the level of data provided here, such as determining cloud base/top height, LWP, *r*_*e*_, and precipitation rate; in these cases the users should exercise their own discretion as to the calculation method to use, how much data along vertical or level-legs to use, and the threshold values of various parameters that are most suitable for their application.

## Additional information

**How to cite this article:** Sorooshian, A. *et al.* A multi-year data set on aerosol-cloud-precipitation-meteorology interactions for marine stratocumulus clouds. *Sci. Data* 5:180026 doi: 10.1038/sdata.2018.26 (2018).

**Publisher’s note:** Springer Nature remains neutral with regard to jurisdictional claims in published maps and institutional affiliations.

## Supplementary Material



## Figures and Tables

**Table 1:** Definitions of acronyms and variables.

**Acronym/Variable Name**	**Definition**
AMS	Aerosol Mass Spectrometer
BC	Black Carbon
BOAS	Biological and Oceanic Atmospheric Study
CAPS	Cloud, Aerosol, and Precipitation Spectrometer
CAS	Cloud and Aerosol Spectrometer
CCN	Cloud Condensation Nuclei
CDP	Cloud Droplet Probe
CIP	Cloud Imaging Probe
CIRPAS	Center for Interdisciplinary Remotely-Piloted Aircraft Studies
CPC	Condensation Particle Counter
C-ToF-AMS	Compact Time-of-Flight Aerosol Mass Spectrometer
CVI	Counterflow Virtual Impactor
DMA	Differential Mobility Analyzer
DMT	Droplet Measurement Technologies
E-PEACE	Eastern Pacific Emitted Aerosol Cloud Experiment
FASE	Fog and Stratocumulus Evolution Experiment
FSSP	Forward Scattering Spectrometer Probe
GPS	Global Positioning System
HiRes-ToF-AMS	High Resolution Time-of-Flight Aerosol Mass Spectrometer
IC	Ion Chromatography
ICP-MS	Inductively Coupled Plasma Mass Spectrometry
ICP-QQQ	Triple Quadrupole Inductively Coupled Plasma Mass Spectrometry
INS	Inertial Navigation System
LPM	Liter Per Minute
LWC	Liquid Water Content
LWP	Liquid Water Path
MASE	Marine Stratus/Stratocumulus Experiment
NiCE	Nucleation in California Experiment
PBAP	Primary Biological Aerosol Particles
PCASP	Passive Cavity Aerosol Spectrometer Probe
PILS	Particle-Into-Liquid Sampler
PMS	Particle Measuring Systems
PSL	Polystyrene Latex Sphere
R/V	Research Vessel
RH	Relative Humidity
SMPS	Scanning Mobility Particle Sizer
SP2	Single Particle Soot Photometer
SST	Skin Surface Temperature
TAS	True Aircraft Speed
UFCPC	Ultrafine Condensation Particle Counter
VOC	Volatile Organic Compound
WIBS	Waveband Integrated Bioaerosol Sensor
*D*_*p*_	Particle Diameter
*N*_*d*_	Cloud Droplet Number Concentration
*r*_*e*_	Cloud Droplet Effective Radius
*T*_*v*_	Virtual Temperature
*θ*	Potential Temperature
*θ*_*e*_	Equivalent Potential Temperature
*θ*_*v*_	Virtual Potential Temperature

**Table 2:** Summary of campaign dates and flight times (Local Time=UTC – 7 h). Data are not available from some or all parts of E-PEACE Flight 3, NiCE Flights 5C and 10C, or FASE Flight 7B

**Campaign**	**Flight #**	**Flight Date**	**UTC: Take Off**	**UTC: Landing**	**Campaign**	**Flight #**	**Flight Date**	**UTC: Take Off**	**UTC: Landing**
MASE I	1	07/02/2005	20:10:35	22:47:03	NiCE	5b	07/12/2013	18:02:41	21:24:01
MASE I	2	07/03/2005	17:03:24	20:18:10	NiCE	5c	07/12/2013	22:01:30	22:52:56
MASE I	3	07/05/2005	16:57:09	21:00:00	NiCE	6	07/15/2013	17:04:04	21:10:07
MASE I	4	07/06/2005	17:03:06	19:53:35	NiCE	7	07/16/2013	17:11:59	21:29:14
MASE I	5	07/08/2005	16:59:14	21:00:02	NiCE	8	07/17/2013	14:57:25	19:41:54
MASE I	6	07/09/2005	17:01:38	20:53:00	NiCE	9	07/18/2013	17:08:19	21:12:22
MASE I	7	07/10/2005	17:01:17	21:18:00	NiCE	10a	07/19/2013	16:11:29	19:21:06
MASE I	8	07/11/2005	18:59:49	22:50:00	NiCE	10b	07/19/2013	20:05:40	0:35:08
MASE I	9	07/13/2005	17:19:26	20:50:00	NiCE	10c	07/19/2013	1:08:57	2:27:20
MASE I	10	07/14/2005	17:32:20	21:17:00	NiCE	11	07/22/2013	17:03:25	21:25:40
MASE I	11	07/15/2005	17:16:14	21:25:00	NiCE	12	07/23/2013	16:39:32	21:36:34
MASE I	12	07/16/2005	17:23:28	21:50:00	NiCE	13	07/24/2013	16:38:32	20:05:59
MASE I	13	07/17/2005	17:01:56	21:22:00	NiCE	14	07/25/2013	15:18:32	19:52:53
MASE II	1	07/10/2007	20:49:54	0:46:36	NiCE	15a	07/26/2013	16:30:10	19:33:43
MASE II	2	07/11/2007	16:59:05	21:25:30	NiCE	15b	07/26/2013	20:44:48	0:27:25
MASE II	3	07/12/2007	18:00:20	22:12:58	NiCE	16	07/29/2013	17:04:03	21:20:51
MASE II	4	07/14/2007	17:21:30	21:21:03	NiCE	17a	07/30/2013	14:49:59	18:19:13
MASE II	5	07/15/2007	18:28:04	21:30:24	NiCE	17b	07/30/2013	19:21:00	22:29:59
MASE II	6	07/16/2007	20:28:30	0:50:34	NiCE	17c	07/30/2013	23:03:30	0:24:51
MASE II	7	07/21/2007	16:30:47	21:02:44	NiCE	18	07/31/2013	14:56:20	19:18:58
MASE II	8	07/22/2007	16:01:18	20:29:48	NiCE	19	08/01/2013	17:15:48	21:32:05
MASE II	9	07/23/2007	15:30:14	20:00:15	NiCE	20a	08/02/2013	14:43:39	17:44:24
MASE II	10	07/24/2007	15:58:34	20:22:00	NiCE	20b	08/02/2013	18:44:27	21:24:52
MASE II	11	07/25/2007	16:26:44	20:50:04	NiCE	20c	08/02/2013	22:07:14	23:45:07
MASE II	12	07/26/2007	15:27:43	19:55:19	NiCE	21	08/05/2013	17:30:11	21:36:22
MASE II	13	07/28/2007	15:49:26	20:14:44	NiCE	22	08/06/2013	17:06:09	21:37:21
MASE II	14	07/29/2007	15:28:08	19:43:28	NiCE	23a	08/07/2013	14:38:34	18:06:09
MASE II	15	07/30/2007	18:00:30	21:56:24	NiCE	23b	08/07/2013	19:22:21	23:55:01
MASE II	16	07/31/2007	15:29:11	19:56:49	BOAS	1	07/02/2015	20:14:18	22:26:10
E-PEACE	1	07/08/2011	20:55:30	23:56:47	BOAS	2	07/06/2015	18:47:15	23:02:50
E-PEACE	2	07/09/2011	16:53:18	21:03:48	BOAS	3	07/07/2015	17:45:22	21:34:05
E-PEACE	3	07/13/2011	16:11:43	20:31:00	BOAS	4	07/08/2015	17:45:42	21:50:47
E-PEACE	4	07/14/2011	16:06:33	20:06:00	BOAS	5	07/09/2015	17:13:50	21:36:35
E-PEACE	5	07/15/2011	16:06:20	19:59:39	BOAS	6	07/10/2015	17:46:14	22:15:21
E-PEACE	6	07/16/2011	16:04:54	20:16:24	BOAS	7	07/13/2015	17:44:31	22:19:53
E-PEACE	7	07/17/2011	16:07:44	20:09:06	BOAS	8	07/14/2015	18:44:17	22:26:18
E-PEACE	8	07/19/2011	16:05:40	20:11:38	BOAS	9	07/15/2015	17:48:42	21:34:58
E-PEACE	9	07/21/2011	16:05:41	20:17:03	BOAS	10a	07/16/2015	15:38:08	19:42:30
E-PEACE	10	07/22/2011	16:04:00	20:19:19	BOAS	10b	07/16/2015	21:25:27	0:09:42
E-PEACE	11	07/23/2011	16:03:11	20:08:41	BOAS	11a	07/17/2015	16:00:24	20:28:00
E-PEACE	12	07/24/2011	16:01:13	20:08:20	BOAS	11b	07/17/2015	21:38:57	1:06:22
E-PEACE	13	07/26/2011	16:03:29	20:07:50	BOAS	12a	07/21/2015	15:44:03	19:32:27
E-PEACE	14	07/27/2011	16:37:18	20:35:02	BOAS	12b	07/21/2015	20:14:40	22:59:10
E-PEACE	15	07/28/2011	17:55:58	21:59:49	BOAS	13a	07/22/2015	15:44:10	19:34:30
E-PEACE	16	07/29/2011	16:37:45	20:43:49	BOAS	13b	07/22/2015	20:09:55	0:06:25
E-PEACE	17	08/01/2011	16:01:46	20:02:30	BOAS	14a	07/23/2015	15:05:30	18:36:55
E-PEACE	18	08/02/2011	15:59:39	20:04:36	BOAS	14b	07/23/2015	19:07:39	22:21:42
E-PEACE	19	08/03/2011	16:04:40	20:02:10	BOAS	15	07/24/2015	16:19:12	19:39:42
E-PEACE	20	08/04/2011	15:59:31	19:58:22	FASE	1	07/18/2016	20:40:25	22:09:22
E-PEACE	21	08/05/2011	17:05:00	21:07:00	FASE	2	07/22/2016	15:23:28	20:31:29
E-PEACE	22	08/08/2011	17:28:50	21:48:03	FASE	3	07/25/2016	19:20:28	1:02:00
E-PEACE	23	08/09/2011	16:36:22	20:41:45	FASE	4	07/26/2016	17:19:02	21:49:04
E-PEACE	24	08/10/2011	16:39:52	20:40:42	FASE	5	07/27/2016	17:00:00	21:21:07
E-PEACE	25	08/11/2011	16:24:20	20:18:10	FASE	6A	07/29/2016	15:45:20	16:41:50
E-PEACE	26	08/12/2011	16:32:17	20:32:17	FASE	6B	07/29/2016	17:31:30	21:38:40
E-PEACE	27a	08/15/2011	15:21:24	19:00:33	FASE	6C	07/29/2016	22:49:10	2:16:04
E-PEACE	27b	08/15/2011	20:01:13	22:46:42	FASE	7A	08/01/2016	17:16:32	18:13:50
E-PEACE	27c	08/15/2011	23:31:54	0:49:31	FASE	7B	08/01/2016	19:13:26	23:23:00
E-PEACE	28a	08/16/2011	15:58:01	17:46:10	FASE	8	08/02/2016	18:01:34	22:53:40
E-PEACE	28b	08/16/2011	18:51:06	22:28:59	FASE	9A	08/03/2016	16:56:40	20:15:50
E-PEACE	28c	08/16/2011	23:11:03	0:32:30	FASE	9B	08/03/2016	21:06:20	0:56:20
E-PEACE	29a	08/17/2011	17:56:30	20:49:14	FASE	10	08/04/2016	17:34:11	23:01:00
E-PEACE	29b	08/17/2011	21:34:34	1:17:51	FASE	11	08/05/2016	16:18:04	19:34:59
E-PEACE	30a	08/18/2011	16:46:50	19:29:48	FASE	12	08/08/2016	17:41:55	22:20:42
E-PEACE	30b	08/18/2011	20:15:32	21:35:49	FASE	13	08/09/2016	17:14:35	21:06:00
NiCE	1	07/08/2013	23:26:20	0:29:03	FASE	14	08/10/2016	16:20:12	21:13:29
NiCE	2	07/09/2013	17:36:51	22:38:08	FASE	15A	08/11/2016	16:12:35	19:31:50
NiCE	3	07/10/2013	15:32:03	19:48:21	FASE	15B	08/11/2016	20:35:40	0:18:00
NiCE	4	07/11/2013	15:18:24	19:35:08	FASE	16	08/12/2016	17:37:50	21:02:42
NiCE	5a	07/12/2013	15:09:01	17:11:38					

**Table 3:** Summary of Twin Otter payload during the six field campaigns.

**Parameter**	**Instrument**	**Diameter Range**	**Time Resolution**	**MASE I**	**MASE II**	**E-PEACE**	**NiCE**	**BOAS**	**FASE**
Aerosol Size Distribution and Particle Concentration	CPC 3010	> 10 nm	1 s	X	X	X	X	X	X
	UFCPC 3025	> 3 nm	1 s	X	X	X	X	X	X
	PCASP	~0.1–2.6 μm	1 s	X	X	X	X	X	X
	SMPS	~ 10–800 nm	~ 75 s	X	X	X	X	X	
Aerosol Composition	PILS coupled to Ion Chromatography	< 1 μm	~5 min	X	X		X		
	AMS	~60–600 nm	< 15 s	X	X	X	X	X	
	SP2	< 1 μm	1 s			X			
Aerosol Hygroscopicity	CCN counter	NA	1 s		X	X	X	X	X
Cloud Drop Size Distribution	CAPS (CASF + CIP)	0.5–1600 μm	1 s	X	X	X	X		X
	FSSP	1–46 μm	1 s	X	X		X	X	X
	CDP	2–52 μm	1 s			X			
Others	Meteorology/Thermodynamics (e.g., T, RH, P, winds)	NA	1 s	X	X	X	X	X	X
	Navigational	NA	1 s	X	X	X	X	X	X
	Light Diffraction (Gerber PVM-100A probe)	NA	1 s	X	X	X		X	X
	Cloud Water Collector	NA	~5-60 min			X	X	X	X
	WIBS-4	0.5–16 μm	1 s					X	
	CO/CO_2_	NA	1 s					X	X
	NO_X_	NA	1 s					X	
	O_3_	NA	1 s					X	
	CVI	NA	NA	X	X	X	X	X	
	Rear-facing Inlet	NA	NA		X				
	Forward-facing inlet	NA	NA	X	X	X	X	X	X

**Table 4:** Summary of instruments that sampled downstream any of three inlets used on the Twin Otter during the six campaigns listed.

**Campaign**	**Inlet Type**	**AMS**	**PILS**	**SMPS**	**CCN**	**CPC 3010**	**UFCPC 3025**	**SP2**	**WIBS-4**
MASE I	Forward-facing Inlet	X	X	X	X	X	X		
	CVI	X	X	Only Flight 12		X			
MASE II	Forward-facing Inlet	X	X	X	X	X	X		
	CVI	X	X			X			
	Rear-facing Inlet	X	X	X	X	X			
E-PEACE	Forward-facing Inlet	X		X	X	X	X	X	
	CVI	X		X	X	X		X	
NiCE	Forward-facing Inlet	X	X	X	X	X	X		
	CVI	X	X	X	X	X			
BOAS	Forward-facing Inlet	X		X	X	X	X		X
	CVI	X		X	X		X		Only Flights 11A, 12A/B, 13A/B
FASE	Forward-facing Inlet				X	X	X		
Instruments without any inlet assignments for a particular campaign were not deployed in that campaign.									
